# The chemical logic of enzymatic lignin degradation

**DOI:** 10.1039/d3cc05298b

**Published:** 2023-12-20

**Authors:** Timothy D. H. Bugg

**Affiliations:** a Department of Chemistry, University of Warwick Coventry CV4 7AL UK T.D.Bugg@warwick.ac.uk

## Abstract

Lignin is an aromatic heteropolymer, found in plant cell walls as 20–30% of lignocellulose. It represents the most abundant source of renewable aromatic carbon in the biosphere, hence, if it could be depolymerised efficiently, then it would be a highly valuable source of renewable aromatic chemicals. However, lignin presents a number of difficulties for biocatalytic or chemocatalytic breakdown. Research over the last 10 years has led to the identification of new bacterial enzymes for lignin degradation, and the use of metabolic engineering to generate useful bioproducts from microbial lignin degradation. The aim of this article is to discuss the chemical mechanisms used by lignin-degrading enzymes and microbes to break down lignin, and to describe current methods for generating aromatic bioproducts from lignin using enzymes and engineered microbes.

## Abstract 2

The article discusses mechanisms used by enzymes that attack the aromatic polymer lignin found in plant biomass.

## Graphical abstract

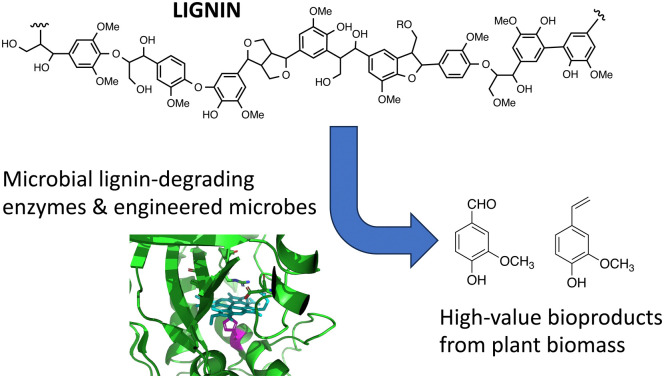

## 1. Introduction

**Fig. 1:**
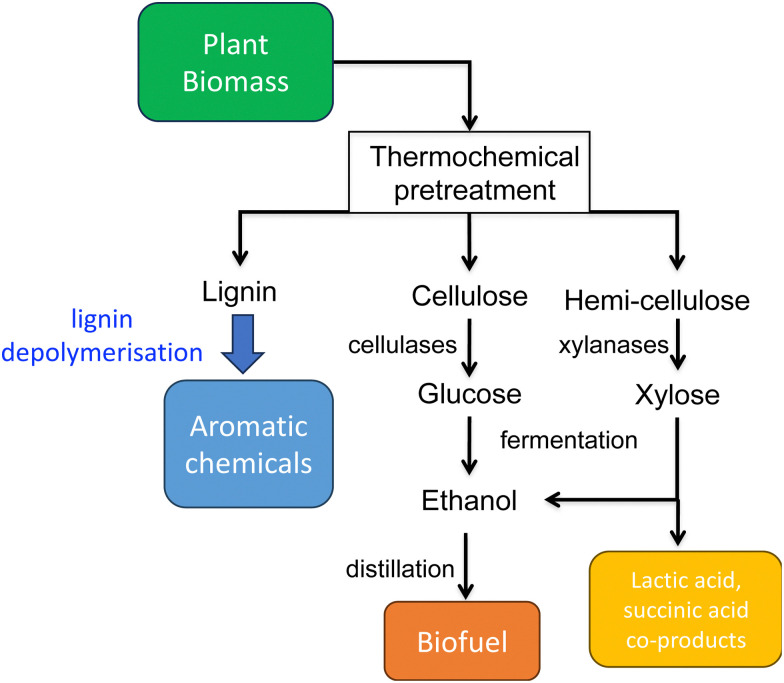
Schematic representation of lignocellulosic biorefinery.

The global impact of the burning of fossil fuels and its effect on climate change has led to a growing realisation that our modern society and its way of life must change to be much more sustainable. For Chemistry, there is also the realisation that much of the chemical industry is built upon the fractionation of crude oil into aliphatic and aromatic chemicals, and their subsequent conversion to feedstock chemicals and plastics. One possible solution is to utilise renewable plant biomass to generate both fuels and chemicals, the “biorefinery” concept (see Fig. 1).^1,2^ Plant cell walls consist mainly of lignocellulose, a material comprising three types of polymer: cellulose, hemi-cellulose, and lignin. While cellulose and hemi-cellulose can be converted *via* saccharification into C6 and C5 sugars, and hence converted *via* fermentation into biofuels such as bioethanol or biobutanol, or into aliphatic carboxylic acids,^1,2^ lignin is a much more refractory polymer, that in pulp/paper and biofuel industries is often burnt to generate power.

However, lignin represents the most abundant source of renewable aromatic carbon in the biosphere, hence, if it could be depolymerised efficiently, then it would be a highly valuable source of renewable aromatic chemicals.^3–5^ Hence the conversion of lignin into renewable chemicals is a key challenge of the lignocellulosic biorefinery concept, but lignin presents a number of difficulties for biocatalytic or chemocatalytic breakdown.^5–7^ Strategies for chemocatalytic lignin conversion have been reviewed.^3–5^ The aims of this article are: to discuss why at the molecular level lignin is so challenging to break down; to explain the interesting chemical mechanisms used by lignin-degrading enzymes and microbes use to break down lignin; and to describe current methods for generating aromatic bioproducts from lignin using enzymes and engineered microbes.

## 2. Chemical structures of polymeric lignin

Lignin is a heteropolymer, made up of aryl-C3 units linked together *via* different types of linkages, involving ether C–O bonds and in some cases C–C bonds, both of which are not susceptible to cleavage using aqueous acid or alkali.^8,9^ The reason for the heterogeneous structure of lignin is that it is biosynthesised in the plant by a radical polymerisation of monolignol precursors (*p*-coumaryl alcohol, coniferyl alcohol, sinapyl alcohol), which can link together in different ways.^8^

**Fig. 2:**
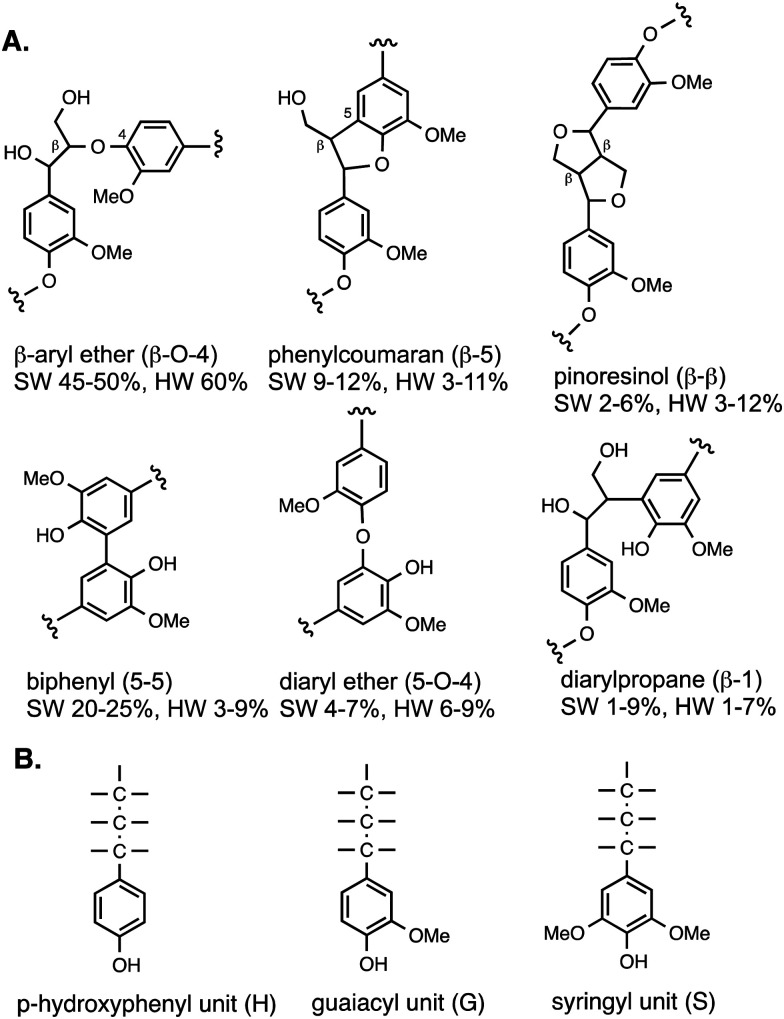
(A) Structures of lignin substructures, and their occurrence in softwood (SW) and hardwood (HW). (B) Structures of H, G, and S units found in polymeric lignin.

The types of linkages found in lignin are illustrated in Fig. 2, which also shows the H, G and S units formed from the three monolignol precursors. Softwood lignin contains predominantly G units, hardwood lignin contains S and G units, while grass lignins contain G, S and H units. The most common type of linkage is the β-O-4 linkage, in which the β-carbon of the C3 sidechain is linked *via* an ether bond to O-4 of the next aryl unit, which comprises 45–60% of the linkages found in native lignin.^8,9^ The biphenyl linkage (5–5) is found as 20–25% of softwood lignin, but much less in hardwood lignin, since the 5-position is substituted in syringyl (S) units. The phenylcoumaran (β-5) linkage and pinoresinol (β–β) linkages both contain C–C bond linkages, and the former also contains a fused dihydrofuran ring. Diaryl ether and diarylpropane units are less common. Although there are several types of linkage present, these same units are found in different types of plant biomass, therefore, depolymerisation strategies should be applicable to different types of plant biomass feedstocks. Grass lignins are also acylated at the γ-position with *p*-hydroxycinnamic acids (*p*-coumaric acid and ferulic acid), which are also attached to hemi-cellulose, and comprise 0.5–4% dry weight of lignocellulose. There are detailed reviews available on lignin structure.^8,9^

There are several different lignin preparations used for lignin depolymerisation studies, whose chemical composition varies considerably. Organosolv lignin is isolated using an organic solvent/organic acid treatment at elevated temperature, which retains much of the structure of native plant lignin,^10^ but has low water solubility, and is not commercially available. Soda lignin is obtained by treatment with sodium hydroxide, followed by acidification and precipitation.^11,12^ Kraft lignin is a by-product of the industrial Kraft process for pulp/paper manufacture, involving treatment with H_2_S.^13^ Although available industrially (and commercially from Sigma-Aldrich), Kraft lignin has a condensed structure resulting from loss of the α-hydroxyl group,^11–13^ which is more difficult to depolymerise (discussed in Section 5), and also contains sulfur, which often poisons chemical catalysts. Lignosulfonate is a by-product of the industrial sulfate process for pulp/paper manufacture, and is also chemically modified, containing sulfonate groups in its chemical structure.^14^ Other types of lignin preparation can be obtained for different biomass pretreatments,^5^ such as ionic liquid treatment,^15^ or using green solvents such as γ-valerolactone.^16^ The chemical structure and solubility of each type of lignin influences its reactivity towards both chemocatalytic and biocatalytic conversion,^17^ which will be discussed in Section 4, but it is important to note that each type of lignin is structurally different, and its reactivity is different.

## 3. Introduction to microbial lignin degraders

**Fig. 3:**
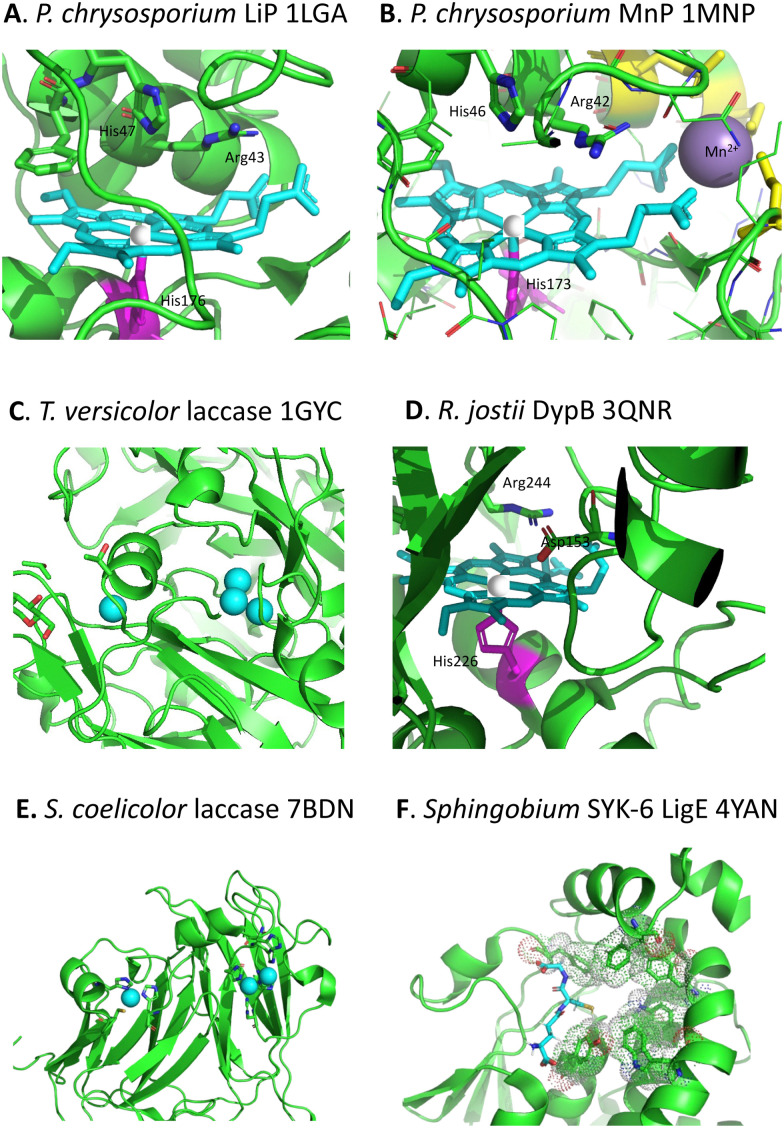
Active sites of lignin-degrading enzymes, with their respective PDB codes. (A) *Phanerochaete chrysosporium* LiP (PDB 1LGA), showing heme cofactor (cyan), Fe centre (brown), catalytic residues His-47, Arg-43, and distal His-176 (magenta). (B) *Phanerochaete chrysosporium* MnP (PDB 1MNP), showing heme cofactor (cyan), Fe centre (brown), bound Mn^2+^ (purple), catalytic residues His-46, Arg-42, and distal His-173 (magenta). (C) *Trametes versicolor* laccase (PDB 1GYC), showing Cu_A_ and trinuclear Cu_B_ centres (cyan). (D) *Rhodococcus jostii* DypB (PDB 3QNR), showing heme cofactor (cyan), Fe centre (brown), catalytic residues His-153, Arg-244, and distal His-226 (magenta). (E) *Streptomyces coelicolor* laccase (PDB 7BDN), showing Cu_A_ and Cu_B_ centres (cyan). (F) *Sphingobium* sp. SYK-6 beta-etherase LigE (PDB 4YAN), showing reduced glutathione substrate (sticks) and non-polar active site residues (spacefill). Images prepared using Pymol software.

Whereas there are many cellulose and hemi-cellulose degraders in the microbial kingdom, the ability to depolymerise lignin is found in only certain specialised microbes. White-rot fungi and brown-rot fungi have the ability to grow on the surface of wood and degrade it, which can be observed in the natural environment.^18,19^ White-rot fungi such as *Phanerochaete chrysosporium* preferentially degrade the lignin fraction of lignocellulose, leaving behind cellulose, which they achieve using extracellular lignin peroxidase (LiP, see Fig. 3A) and manganese peroxidase (MnP, see Fig. 3B) enzymes, and multi-copper oxidase or laccase enzymes (see Fig. 3C).^18,19^ Brown-rot fungi such as *Postia placenta* chemically modify the lignin structure using biological Fenton chemistry to generate hydroxyl radical,^20^ but this is used to disrupt the lignocellulose structure so that the microbe can preferentially attack the cellulose and hemi-cellulose polysaccharides, leaving behind the modified lignin residue.

Although there were reports in the 1980s that certain bacteria such as *Streptomyces viridosporus* could depolymerise lignin,^21^ no gene or enzyme responsible was identified until 2011, when a dye-decolorizing peroxidase DypB was identified in *Rhodococcus jostii* RHA1 (see Fig. 3D).^22^ Further dye-decolorizing peroxidases active for lignin degradation have also been identified in *Amycolatopsis* sp. 75iv2,^23^*Pseudomonas fluorescens*,^24^ and *Thermobifida fusca*.^25^ Several *Streptomyces* soil bacteria contain small laccase enzymes that can attack lignin (see Fig. 3E),^26^ and multi-copper oxidase enzymes active for lignin degradation have been identified in *Ochrobactrum* sp.,^27^*Pseudomonas putida* and *Pseudomonas fluorescens*.^28^ Several soil bacteria with the ability to degrade aromatic compounds have also been shown to degrade lignin,^29,30^ of which the best developed microbes for metabolic engineering studies are *Rhodococcus jostii* RHA1 and *Pseudomonas putida* KT2440, though other microbes also have the potential to be used as microbial hosts for lignin degradation.^31^ Analysis of the genomes of lignin-degrading bacteria reveals that nearly all contain either dye-decolorizing peroxidases or multi-copper oxidases, or both, and that most contain the β-ketoadipate pathway for metabolism of protocatechuic acid, a key intermediate in lignin breakdown.^32^

## 4. Mechanisms for difficult bond cleavages

### 4.1. Cleavage of β-O-4 ether bond

The β-aryl ether (β-O-4) linkage is the most common type of linkage found in polymeric lignin, and is the linkage most frequently cleaved by lignin degradation enzymes. Experimental studies have shown a correlation between lignin β-O-4 content and conversion to low molecular weight products for enzymatic and chemocatalytic processes,^17,33^ hence high β-O-4 content is a desirable property for lignin valorisation.

Chemocatalytic mechanisms for cleavage of the β-aryl ether linkage fall into the following classes:^5^ (1) base-catalysed cleavage reactions *via* quinone methide intermediates; (2) acid-catalysed reactions *via* benzylic carbocation intermediates; (3) reductive cleavage *via* high pressure hydrogenation with transition metal catalysts to give alkyl sidechain products; (4) oxidative cleavage to give products containing oxidative sidechains. Enzymes, which operate close to pH 7, do not employ strategies 1–3 above, and the types of enzyme used by lignin-degrading microbes mainly catalyse oxidative reactions. The most common enzymatic mechanism for β-aryl ether cleavage involves oxidative Cα–Cβ cleavage.

Studies using model β-aryl ether lignin dimer substrates have shown that Cα–Cβ oxidative cleavage to form vanillin is observed for fungal LiP enzymes^18^ and for bacterial *R. jostii* DypB.^22^ The fungal LiP is able to cleave substrates containing an alkylated C-4 hydroxyl group (or non-phenolic unit),^18^ due to its higher redox potential, whereas DyP enzymes usually require a free C-4 hydroxyl group.^22^ Both the fungal LiP^34^ and bacterial DyP^22^ enzymes react with hydrogen peroxide to form a heme iron–oxo intermediate (known as compound I), which is able to carry out one-electron oxidation reactions. As shown in Fig. 3, in fungal LiP the distal face of the heme cofactor, where the iron–oxo intermediate is formed, contains nearby histidine and arginine residues,^35^ whereas in bacterial DyP there are nearby aspartate and arginine residues on the distal heme face.^36^

**Fig. 4:**
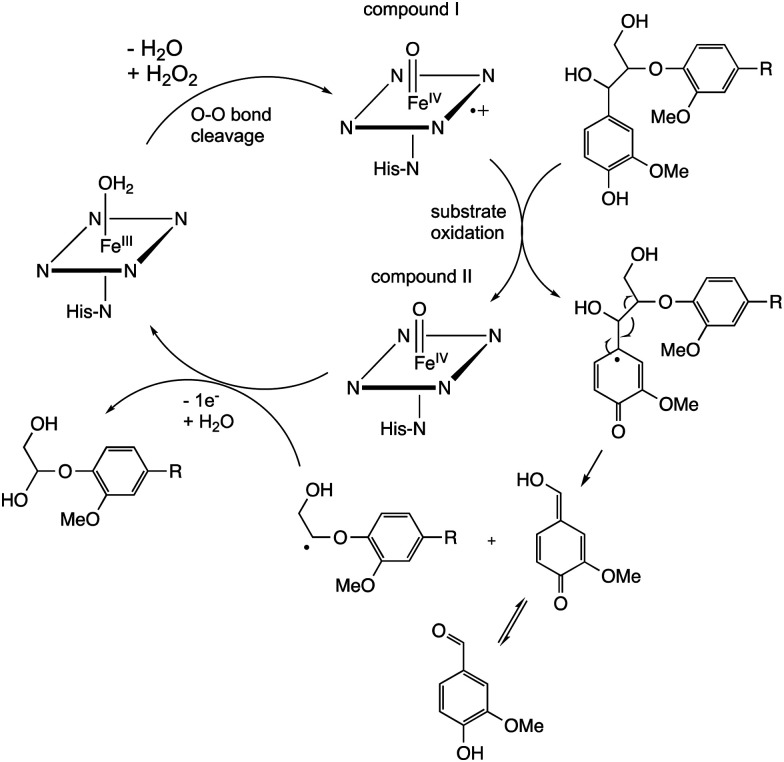
Mechanism of Cα–Cβ bond cleavage *via* radical mechanism.

The mechanism of Cα–Cβ bond cleavage is proposed to take place *via* a radical mechanism, where either a phenoxy radical (for DyPs^22^) or an aryl radical cation (for LiP^37^) is formed, bearing radical density at C-1. Elimination at the aryl–Cα bond then cleaves the Cα–Cβ bond, as shown in Fig. 4. There is a possible alternative mechanism involving further oxidation to a quinone methide intermediate,^5^ followed by two-electron C–C fragmentation to give an oxonium ion, but evidence has recently been presented against such a mechanism.^38^

**Fig. 5:**
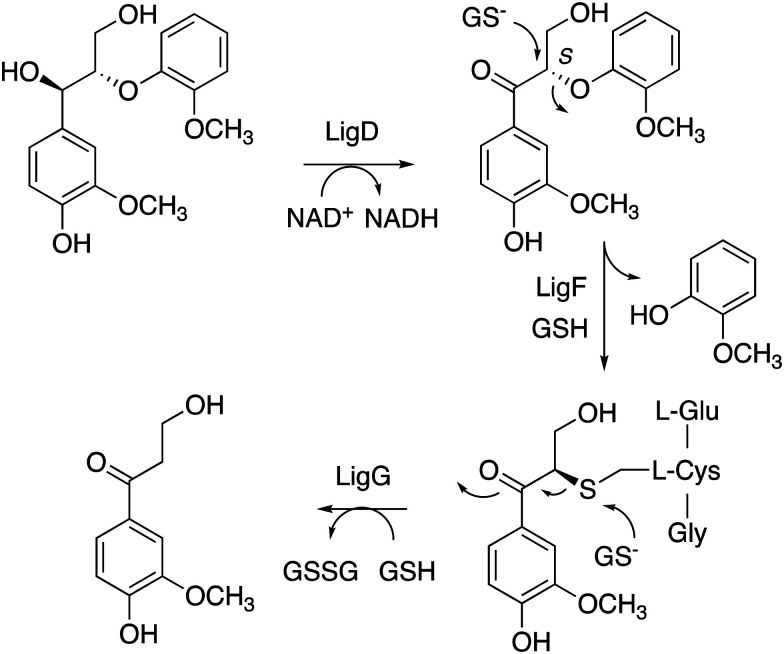
Mechanism of LigFG β-aryl ether cleavage. GSH, reduced glutathione; GSSG, oxidised glutathione. β-Etherase LigE catalyses a similar reaction to LigF, on the *R* enantiomer.

Nucleophilic cleavage of the β-aryl ether linkage is also observed biologically, in the case of the β-etherase enzymes found in *Sphingobium* SYK-6 (see Fig. 3F),^39–42^ and in *Novosphingobium* sp.^43^ These enzymes operate primarily on lignin dimers, which are broken down by these bacteria.^39–43^ An initial oxidation of the α-hydroxyl group takes place, catalysed by dehydrogenase LigD.^44^ The presence of a benzylic ketone enhances the electrophilicity of the β-ether bond. Two β-etherase enzymes LigE and LigF then catalyse stereospecific cleavage of the β-ether bond,^41,42^ using the thiol group of glutathione as a nucleophile, to generate a glutathione conjugate, from which glutathione is removed by enzyme LigG to generate the ketone product (Fig. 5).^40^ Crystal structures of *Sphingobium* SYK-6 LigE and LigF have been determined, identifying the site of glutathione binding, and a non-polar binding site for the lignin dimer substrate,^45^ shown in Fig. 3. The use of glutathione for β-aryl ether cleavage has been mimicked chemically *via* use of thiols to carry out reductive cleavage of lignin model compounds and polymeric lignin.^46^

**Fig. 6:**
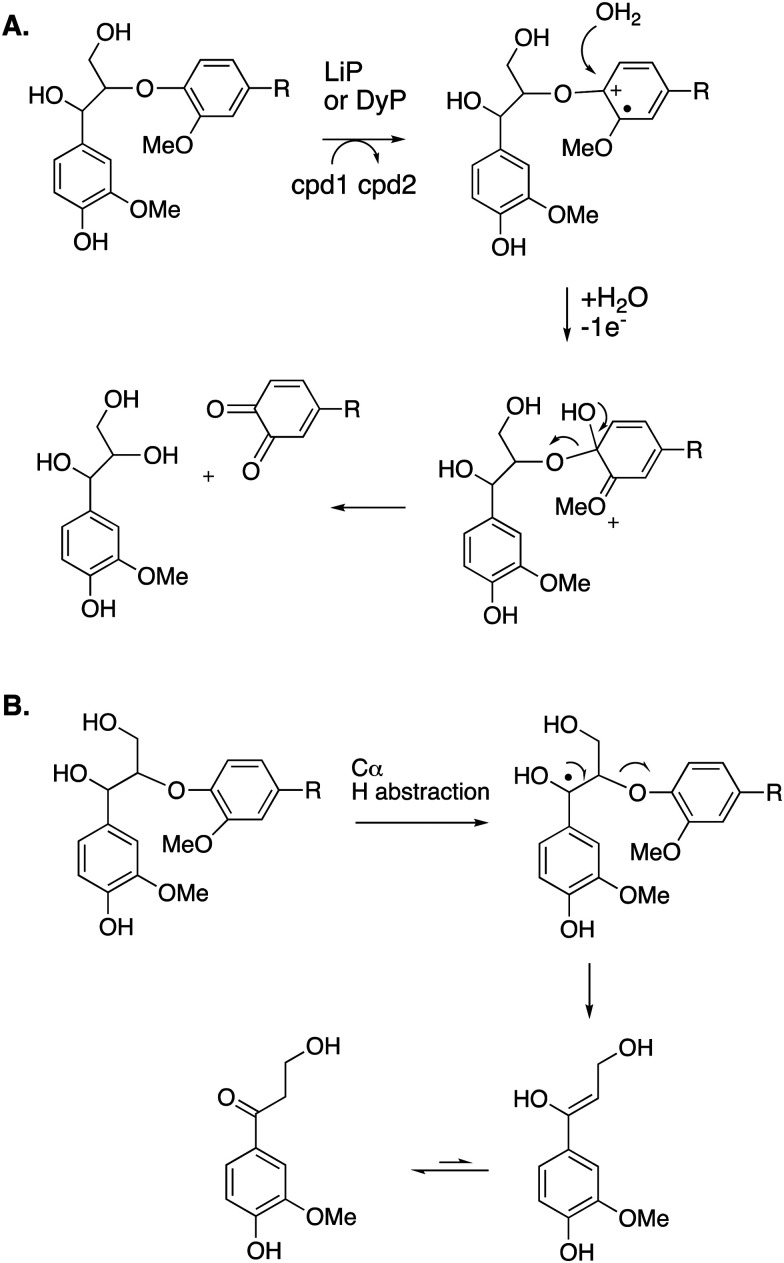
Other possible mechanisms for β-O-4 cleavage, *via* distal C–O cleavage (panel A), or radical elimination (panel B). Compounds 1 and 2 (cpd1, cpd2) are shown in Fig. 4. Electron acceptor for 2nd step would be compound 2.

Cleavage of the distal aryl-O bond of the β-aryl ether linkage to generate aryl C_3_ products containing a β-hydroxy group has been observed in fungal lignin peroxidase using lignin model compounds,^47^ and has been observed by NMR spectroscopy during lignin processing by white-rot fungus C*eriporiopsis subvermispora*.^48^ Related aryl C_3_ products containing either a C_3_ triol sidechain,^17^ or an oxidised ketodiol sidechain,^24^ have been obtained from treatment of polymeric lignin by *P. fluorescens* DyP1B peroxidase.^17,24^ In these cases a probable mechanism would involve hydroxylation of the distal phenolic ring, followed by C–O cleavage, as shown in Fig. 6. This mechanism is supported by ^18^O labelling studies carried out on the processing of lignin dimer model compounds by fungal lignin peroxidase by Gold and co-workers,^47^ showing that the β-hydroxy group of the product is derived from the ether oxygen of the substrate, hence that distal C–O bond cleavage had taken place.

The 3′-hydroxypropiophenone product formed by β-etherase cleavage has also sometimes been reported as a reaction product from oxidative DyP reactions.^49,50^ A radical-based mechanism has been proposed (see Fig. 6B), involving formation of a radical at the α-position, followed by Cα–Cβ elimination to give an enol product, which tautomerises to the corresponding ketone.

### 4.2. Strategies for aryl–Cα cleavage

**Fig. 7:**
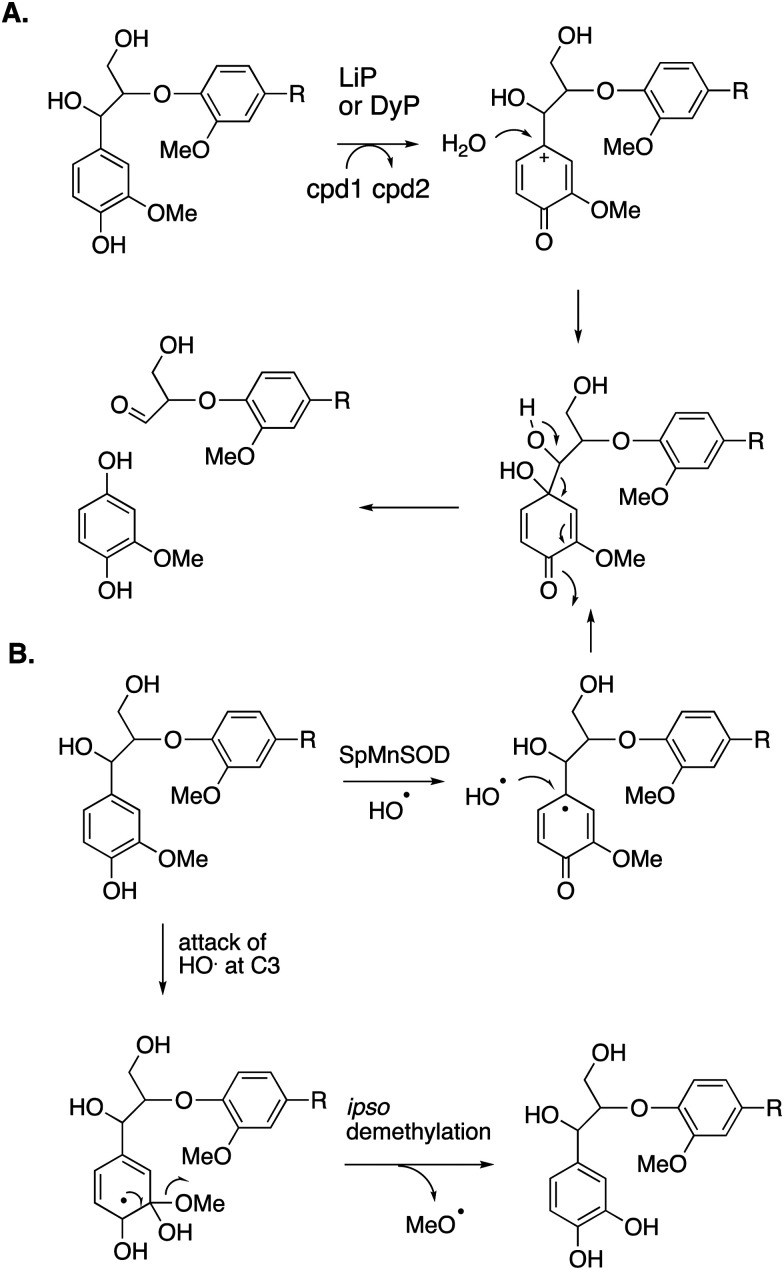
Mechanisms for aryl–Cα cleavage, by fungal LiP or bacterial DyP (panel A); or by *Sphingobacterium* sp. T2 SpMnSOD (panel B), also showing mechanism for demethylation. Compounds 1 and 2 (cpd1, cpd2) are illustrated in Fig. 4.

Reaction products arising from aryl–Cα cleavage of lignin dimer substrates have also been observed from fungal manganese peroxidase,^51^ and bacterial DyPs,^49^ generating products 2,6-dimethoxy-hydroquinone, 2-methoxy-hydroquinone and hydroxyquinol. ^18^O labelling studies using fungal MnP have established that in this case the oxygen atom introduced onto the aromatic ring is derived from water, hence a mechanism involving oxidation of the phenolic ring to an aryl radical cation has been proposed, shown in Fig. 7A.^51^

2-Methoxyhydroquinone has also been observed as a cleavage product from an unusual manganese superoxide dismutase enzyme from *Sphingobacterium* sp. T2, which is able to depolymerise and solubilise lignin.^52^ A mechanism involving generation of hydroxyl radical was proposed,^52^ and the formation of hydroxyl radical was subsequently confirmed.^53^ A mechanism for formation of 2-methoxyhydroquinone is shown in Fig. 7B, *via* attack of hydroxyl radical, followed by retro-aldol cleavage,^52,53^ which is supported by ^18^O labelling studies.^53^ The active site structure of this enzyme is very similar to that of *E. coli* MnSOD, which does not generate hydroxyl radical,^52^ but two amino acid mutations close to the active site were found to be required for the lignin depolymerisation activity, which appear to allow solvent access to the Mn cofactor.^53^ This enzyme also catalyses oxidative demethylation of lignin,^53^ shown to occur *via* an *ipso*-substitution mechanism by hydroxyl radical in which methanol is lost, shown in Fig. 7B. Hydroxyl radical is generated *via* Fenton chemistry in brown-rot fungi,^20^ and has also been detected in white-rot fungi growing on wood.^54,55^ White-rot fungi are also reported to generate methanol from lignin breakdown.^56^ Hence there is more than one strategy in Nature to generate the powerful oxidant hydroxyl radical to attack the recalcitrant polymer lignin.

### 4.3. Cleavage of substructures containing C–C bonds

**Fig. 8:**
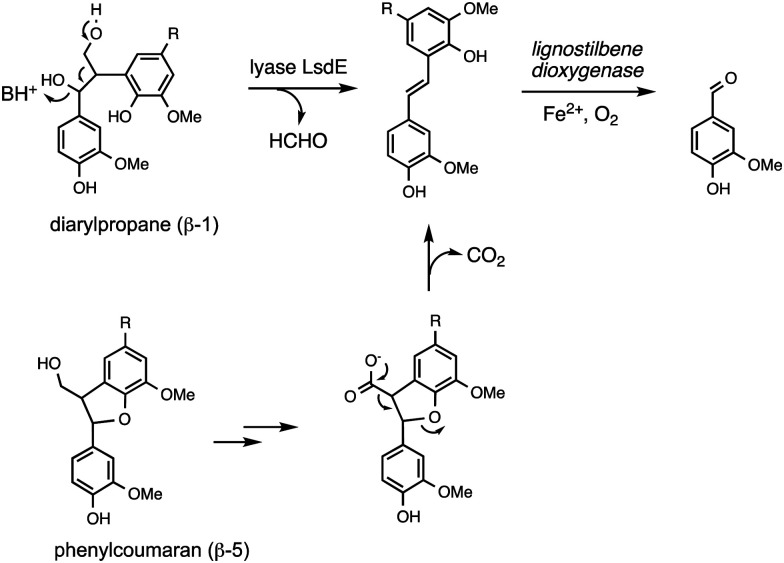
Degradation of diarylpropane (β-1) unit and pinoresinol (β–β) unit *via* lignostilbene.

Oxidative cleavage of diarylpropane (β-1) lignin model compounds by fungal lignin peroxidase has been reported, *via* Cα–Cβ oxidative cleavage,^57^ likely *via* a similar one-electron mechanism to that illustrated in Fig. 4. This reaction was a key piece of evidence for the involvement of radical mechanisms in lignin peroxidase reactions.^57^ A bacterial pathway for degradation of diarylpropane dimers has recently been elucidated in *Novosphingobium aromaticivorans*, by a novel lyase enzyme LsdE, which generates lignostilbene, with formaldehyde as a by-product, as shown in Fig. 8.^58^ The crystal structure of this lyase enzyme has recently been determined, providing some insight into the mechanistic details for this unusual fragmentation reaction.^59^

The phenylcoumaran (β-5) lignin fragment also contains a C–C bond at the β position. A β-5 lignin dimer is metabolised by *Sphingobium* SYK-6 *via* oxidation of the γ-alcohol functional group to a carboxylic acid, followed by decarboxylation, generating a stilbene intermediate, as found on the β-1 degradation pathway above (see Fig. 8), which is then converted to vanillin by non-heme iron-dependent lignostilbene dioxygenase.^60^ The pinoresinol (β–β) unit also contains a C–C bond at the β position, and two tetrahydrofuran rings. Studies of the bacterial degradation of a β–β lignin dimer has shown that in *Sphingobium* SYK-6 reductive cleavage of the tetrahydrofuran ring takes place,^61^ but in *Pseudomonas* sp. SG-MS2, hydroxylation of the α-position occurs, followed by opening of a tetrahydrofuran ring to form a benzylic ketone.^62^ The biphenyl unit found in lignin is thought to be degraded *via* oxidised metabolite 5,5′-dehydrodivanillic acid (DDVA), for which a specialised aromatic degradation pathway has been elucidated in *Sphingobium* SYK-6.^63^ Oxidative cleavage of both DDVA and a β–β lignin dimer was observed using *Sphingobacterium* sp. T2 manganese superoxide dismutase.^53^

### 4.4. Anaerobic degradation of lignin

As described above, the microbes known to degrade lignin are aerobic organisms, that use mainly oxidative enzymes to attack lignin. However, there are some reports of lignin degradation by anaerobic bacteria, such as *Enterobacter lignolyticus*^64^ and *Dysgonomonas*,^65^ and facultative anaerobic bacteria *Agrobacterium* sp., *Lysinibacillus sphaericus*, *Comamonas testosteroni*, and *Paenibacillus* sp.,^66^ the latter organisms enhancing gas release from lignocellulose under anaerobic conditions.^66^ The molecular mechanisms of anaerobic lignin degradation are not well understood, but it has been shown that demethylation of a β-aryl ether lignin dimer occurs,^66^ hence it is possible that the one-carbon unit released is ultimately converted to methane. One possible way that this could happen would be *via* a redox-neutral demethylation, similar to demethylase enzyme LigM which has been identified in *Sphingobium* SYK-6, that uses a tetrahydrofolate coenzyme.^67^Table 1 summarises the different lignin-degrading enzymes discussed, and the types of bond cleavages observed.

**Table 1:** Lignin-degrading enzymes discussed in the text, producing organism, and type of cleavage mechanism observed. MCO, multi-copper oxidase

Microbe	Enzyme	Cleavage mechanisms	Ref.
White rot fungi			
*Phanerochaete chrysosporium*	Lignin peroxidase	Cα–Cβ, Cα ox	18 and 19
	Mn peroxidase	Cα–Cβ	47 and 51
	Laccase	Cα ox, aryl–Cα	57

Bacteria			
*Rhodococcus jostii*	Peroxidase DypB	Cα–Cβ	22
*Pseudomonas fluorescens*	Peroxidase Dyp1B	Cα–Cβ, aryl–Cα	17 and 24
	MCO CopA		28
*Amycolatopsis* sp.	Peroxidase Dyp2		
*Streptomyces coelicolor*	MCO SLAC		23
			26
*Ochrobactrum* sp.	MCO CueO		27
*Sphingobacterium* sp.	SpMnSOD	Aryl–Cα, demethylation	27, 52 and 53

## 5. Physical access to lignin polymer

Besides difficult bond cleavages, there are several other problems that must be solved in order to break down lignin, which will be discussed in turn. Lignin is a polymer, that in the plant cell wall is interwoven with cellulose and hemi-cellulose and insoluble, and in the case of extracted lignin often has low water solubility. This presents several problems to the lignin-degrading microbe. Firstly, the initial phase of lignin oxidation must be extracellular, and therefore, lignin-oxidising enzymes must be exported to the cell surface. White-rot fungi such as *Phanerochaete chrysosporium* produce extracellular lignin peroxidases and laccases to attack lignin.^18^ In bacteria, multi-copper oxidases are generally extracellular, using a TAT signal sequence,^32^ while some DyPs are extracellular,^32^ and some such as *R. jostii* RHA1 DypB are targeted to an encapsulin nanocompartment,^68^ but it is not clear whether such a nanocompartment is exported.

**Fig. 9:**
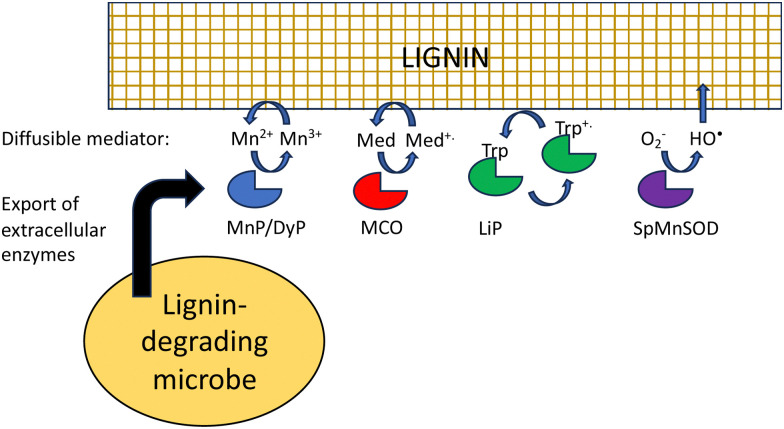
Strategies to provide physical access for oxidants to lignin polymer. LiP, fungal lignin peroxidase; MnP, fungal manganese peroxidase; DyP, bacterial dye-decolorizing peroxidase; MCO, multi-copper oxidase; SpMnSOD, *Sphingobacterium* sp. T2 manganese superoxide dismutase; Med, mediator; Trp, surface tryptophan residue.

The next problem is how does a lignin peroxidase or multi-copper oxidase bind a lignin polymer at its active site? Enzymes that bind polysaccharide substrates, such as lysozyme, generally have solvent-exposed, cleft-like active sites, whereas peroxidases usually have rather narrow active site entrances. It is thought that for fungal manganese peroxidases, the Mn^3+^ oxidation product diffuses into the lignin (or lignocellulose) structure, acting as a diffusible oxidant or mediator (Fig. 9).^18,69^ The bacterial DyPs that show activity for polymeric lignin oxidation also usually catalyse Mn^2+^ oxidation,^22–24^ and lignocellulose breakdown is reported to be dependent on the presence of Mn^2+^.^22,24^

Laccases or multi-copper oxidases can also utilise small molecule mediators, which are diffusible and change the redox potential of the oxidant, both of which change the interaction with polymeric lignin.^70^ For *in vitro* applications, mediators are synthetic reagents such as 2,2′-azino-bis(3-ethylbenzthiazoline-6-sulfonate) (ABTS) or 1-hydroxybenzotriazole (HBT),^71^ which are able to undergo one-electron redox chemistry, but lignin-derived compounds such as syringaldehyde and acetosyringone are also efficient laccase mediators,^72^ that may function *in vivo* in lignin degradation.

Hydroxyl radical is also a low molecular weight oxidant that can also attack lignin. It is generated by Fenton chemistry in brown-rot fungi,^20^ but is also produced in white-rot fungi,^54,55^ and is generated by *Sphingobacterium* sp. T2 manganese superoxide dismutase that oxidises lignin.^52,53^

Fungal lignin peroxidase also utilises a novel strategy to oxidise polymeric lignin. Through an electron transport pathway in the protein structure, a surface tryptophan residue (Trp-171 in *Coprinus cinerius* LiP) is oxidised to a stable tryptophan radical, which can then interact with the lignin polymer.^73^ A surface tryptophan radical has also been reported in *Auricularia auricula-judae* DyP,^74^ hence this strategy may also be utilised by DyP enzymes.

## 6. Condensed lignins are even more difficult to break down

**Fig. 10:**
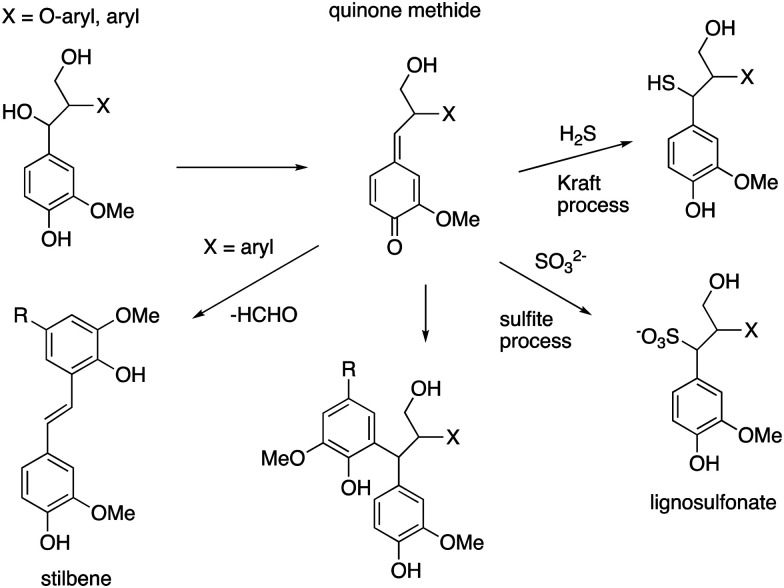
Structures of condensed units found in Kraft lignin and lignosulfonate, formed from β-aryl ether units (X = O-aryl) and diarylpropane units (X = aryl).

Lignin is produced industrially as a by-product of the pulp/paper industry, so it would desirable to use such lignin for conversion to renewable chemicals, however, the industrial (or technical) lignins are more difficult to break down. The two major types are Kraft lignin, from the industrial Kraft process involving treatment with H_2_S,^13^ and lignosulfonate, a by-product of the industrial sulfate process.^14^ They both contain “condensed” structures resulting from loss of the α-hydroxyl group to form quinone methide intermediates, and further reaction to form eliminated stilbenes and diarylmethane structures shown in Fig. 10.^11,14,75^ Although structural analysis of condensed lignins is challenging, partial structures have been observed by detailed NMR spectroscopic studies.^11,75^ The low β-O-4 content of these lignins renders them more difficult to depolymerise,^17^ and they also contain sulfur, which tends to poison chemical catalysts.

It is therefore interesting that some microbes are able to convert Kraft lignin to useful products. The ability of a microbe to grow on industrial Kraft lignin does not in itself prove that it is breaking down polymeric lignin, because Kraft lignin also contains low molecular weight aromatics, which are much more readily broken down, however, in some cases it is clear that lignin depolymerisation is taking place. Several oleaginous lignin-degrading bacteria are able to accumulate triacylglycerol lipids as internal carbon storage compounds, and *Rhodococcus* sp. have been extensively studied due to their capacity of accumulating high amounts of lipids from lignin and lignin-derived aromatics. *R. opacus* DSM 1069 was reported to accumulate 67 mg L^−1^ of TAGs after 36 h growth from oxygen-pretreated Kraft lignin.^76^ Further examples of conversion of Kraft lignin to aromatic products will be mentioned in Section 10. The mechanisms by which condensed lignin units are broken down are not known, but the use of combinations of lignin-oxidising enzymes and accessory enzymes (discussed in Sections 7 and 8) has been reported to be more effective for release of low molecular weight products from technical lignins.^49^

## 7. Repolymerisation of phenoxy radical intermediates

The major product often obtained from treatment of lignin model compounds *in vitro* with purified lignin-oxidising enzymes such as lignin peroxidase, DyPs and multi-copper oxidases is, surprisingly, higher molecular weight phenolic material!^18,22^ The explanation is that radical depolymerisation leads to phenoxy radical intermediates, which repolymerise to give higher molecular weight products, similar to lignin biosynthesis in plants. This is a formidable challenge in the use of recombinant enzymes for lignin bioconversion *in vitro.* But as noted by Kirk and Farrell in the review of microbial lignin oxidation in 1987, this phenomenon is generally not observed in microbial lignin conversion,^18^ therefore, there must be mechanisms to prevent repolymerisation *in vivo*.

**Fig. 11:**
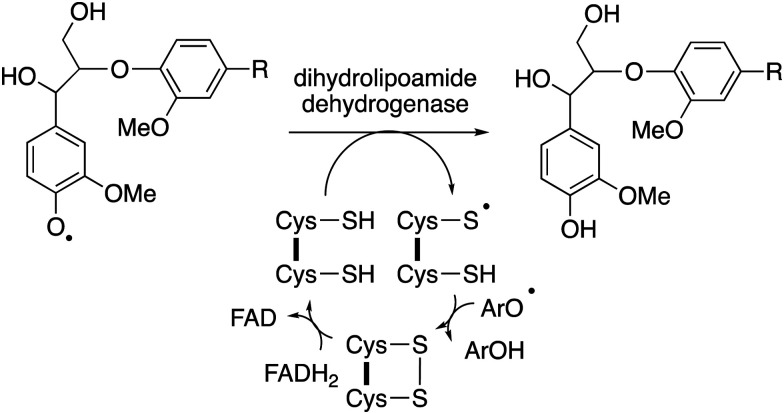
Mechanism of phenoxy radical trapping by FAD-dependent dihydrolipoamide dehydrogenase containing an active site disulfide. ArOH represents a second 1-electron reduction of a phenoxy radical to a phenol.

An accessory enzyme capable of one-electron reduction of phenoxy radicals to the corresponding phenol could in theory prevent such a reaction. A highly expressed extracellular dihydrolipoamide dehydrogenase from *Thermobifida fusca* has been identified, which can prevent the dimerization of a lignin model compound *in vitro*, and to alter the profile of low molecular weight products produced.^77^ Combinations of bacterial DyPs and bacterial dihydrolipoamide dehydrogenase enzymes have been shown *in vitro* to generate enhanced yields of low molecular weight aromatic products, and new types of products.^49^ This enzyme is a disulfide reductase containing an active site cysteine disulfide and a flavin adenine dinucleotide cofactor, which facilitates a one-electron reduction mechanism,^77^ as shown in Fig. 11. In fungi, the enzyme cellobiose dehydrogenase is believed to fulfil this role *in vivo*, this is a flavin-dependent enzyme also containing a heme cofactor (cytochrome domain).^78^ This enzyme has been reported to be synergistic with fungal manganese peroxidase for degradation of Kraft pulp lignin.^79^

Redox protein peroxiredoxin from *Burkholderia cepacia* has also been reported as an effective accessory protein, in combination with bacterial DyPs.^49^ Peroxiredoxin has been observed in proteomic analysis of lignin-degrading *Pandoraea*,^80^*Bacillus ligniniphilus* L1,^81^ and *Sphingobacterium* sp. T2.^52^ This protein reacts with hydrogen peroxide through an active site cysteine nucleophile, to form a sulfenic acid intermediate (sidechain CH_2_S–OH).^82^ Sulfenic acids are known to act as potent radical scavengers,^83^ and are released from alliin, the active ingredient in garlic,^83^ hence it is possible that peroxiredoxin could also scavenge phenoxy radical intermediates in lignin degradation.

## 8. Generation of hydrogen peroxide co-substrate

Hydrogen peroxide is needed as the co-substrate for fungal lignin peroxidase and bacterial DyP peroxidases, leading to the question of what is the source of hydrogen peroxide *in vivo*? In fungi, several extracellular oxidase enzymes have been characterised that can generate extracellular hydrogen peroxide,^84^ of which aryl alcohol oxidase^85^ and glyoxal oxidase^86^ are thought to be involved in lignin breakdown. Aryl alcohol oxidase from *Pleurotus eryngii* is able to oxidise benzylic alcohols efficiently,^87^ which are generated from lignin breakdown, and this oxidation has been shown to generate hydrogen peroxide extracellularly in *Pleurotus eryngii*.^88^ Glyoxal oxidase from *Phanerochaete chrysosporium* is able to oxidise aliphatic aldehydes, α-hydroxycarbonyl compounds and α-dicarbonyl compounds,^89,90^ which are possible oxidation products of the C_3_ alkyl sidechain of lignin, and has been shown to support hydrogen peroxide generation extracellularly in *P. chrysosporium*.^89^ Two further glyoxal oxidases have been characterised from *Pycnoporus cinnabarinus*.^91^*P. eryngii* aryl alcohol oxidase has been reported to work effectively in combination with fungal lignin peroxidase *in vitro*.^92^

In bacteria, an FMN-dependent glycolate oxidase has been identified that participates in a 4-hydroxybenzoylformate degradation pathway in *Rhodococcus jostii* RHA1, thought to degrade aryl C_2_ fragments from lignin degradation.^93^ This glycolate oxidase oxidises a range of aromatic and aliphatic α-dicarbonyl compounds and α-hydroxycarboxylic acids, and is reported to function effectively in combination with bacterial DyPs *in vitro*, generating enhanced yields of low molecular weight products from polymeric lignin oxidation, and novel products.^50^ This combination was also effective for oxidation of biorefinery lignins and polymeric humins.^50^ A copper-dependent oxidase from *Thermobifida fusca* has been reported that enhances lignin breakdown in sugarcane bagasse,^94^ and a pyranose 2-oxidase from *Kitasatospora aureofaciens* has been reported that couples effectively with manganese peroxidase *in vitro*.^95^ An advantage of using oxidase–peroxidase combinations is that peroxidase enzymes are often inactivated by millimolar concentrations of hydrogen peroxide, therefore, the controlled release of low concentrations of hydrogen peroxide by an oxidase enzyme lengthens the lifetime of the biocatalyst, and therefore improves conversion yield.^50^

## 9. Generation of toxic intermediates

A further complication of lignin breakdown is the generation of reactive or toxic intermediates that could either act as enzyme inhibitors or microbial growth inhibitors. Lignin-derived phenolic compounds generated from biomass pretreatment are known to act as enzyme inhibitors for cellulase enzymes used for saccharification of cellulose,^96^ hence there is a need for strategies to remove or mitigate such phenolic inhibitors during cellulosic biofuel production.^96–98^ One advantage of microbial hosts that are degraders of phenolic compounds is that they have evolved resistance mechanisms against such phenolic compounds.^99,100^

**Fig. 12:**
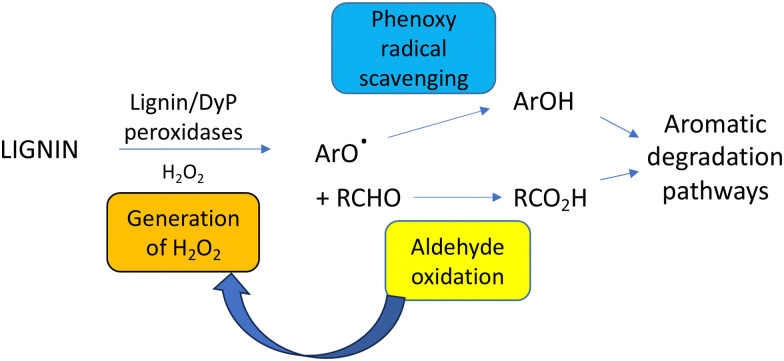
Roles of accessory enzymes in lignin degradation.

Phenolic and furanic aldehydes are also generated from lignin pretreatment, which can function as enzyme inhibitors.^96^ Microbial lignin degradation generates phenolic aldehydes vanillin and syringaldehyde as oxidation products, which are degraded by aromatic degradation pathways *via* the corresponding benzoic acids.^101^ Oxidation of the C_3_ alkyl sidechain found in lignin generates aldehydes such as glyoxal and methylglyoxal, which are oxidised by the fungal glyoxal oxidase^86^ and the bacterial glycolate oxidase.^50^ Hence, an advantage of using such oxidase enzymes in combination with lignin-oxidising peroxidases is that each enzyme generates the substrate for the other enzyme, as shown in Fig. 12.^50^ Oxalic acid is often detected as a metabolite from microbial lignin breakdown, and it has been shown that the bacterial glycolate oxidase can catalyse three successive oxidations of glycolaldehyde, released from β-aryl ether oxidative cleavage, to oxalic acid.^38^

## 10. Use of engineered microbes for generation of bioproducts from lignin degradation

The final challenge in the conversion of lignin to target chemicals is the generation of complex mixtures of products. Polymeric lignin is itself heterogeneous in structure, as described in Section 2. Processing of a heterogeneous substrate by non-specific enzymatic oxidation, or chemocatalytic processing, or other methods such as pyrolysis, is therefore inevitably going to lead to complex mixtures of low molecular weight products, for which analytical methods are being developed to deconvolute and structurally characterise.^102,103^ Given the practical limitations of using lignin-oxidising enzymes *in vitro* mentioned in Sections 7–9, the use of enzyme combinations for lignin processing *in vitro* is an approach currently being explored,^50,104,105^ but mixtures of products are still generated.

**Fig. 13:**
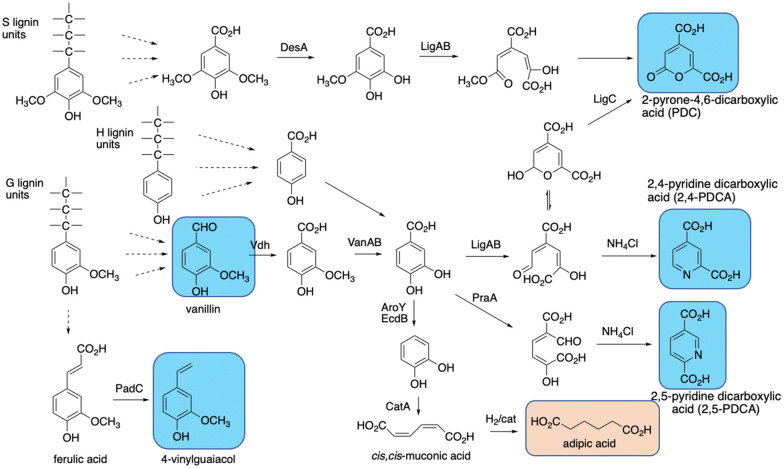
Production of bioproducts from lignin using engineered recombinant bacterial strains. Aromatic products shown in blue, non-aromatic products shown in brown.

The use of microbial degraders to process such mixtures has an advantage that aromatic degradation is convergent, funnelling multiple substrates to a limited number of common intermediates such as vanillic acid and protocatechuic acid, which are then metabolised *via* common pathways, such as the β-ketoadipate pathway.^101,106,107^ Therefore, metabolic engineering of aromatic degradation pathways can lead to selective bioproduct formation. Examples of such bioproducts are shown in Fig. 13. In 2013, Sainsbury *et al.* reported that growth of a *Rhodococcus jostii* Δ*vdh* gene deletion strain in 2.5% milled wheat straw led to an accumulation of 96 mg L^−1^ of vanillin after 6 days, in addition to 53 mg L^−1^ 4-hydroxy-benzaldehyde,^108^ consistent with the degradation of both G- and H-lignin units present in wheat straw lignocellulose (see Fig. 12). Later studies showed that this gene deletion strain could accumulate 243 mg L^−1^ syringaldehyde from hardwood organosolv lignin as feedstock, *via* processing of the S-lignin units found in hardwood lignin.^17^

*cis,cis*-Muconic acid has been generated in high titre in engineered *Pseudomonas putida* KT2440 strains, *via* insertion of decarboxylase *aroY* which converts protocatechuic acid to catechol, and gene knockout of *pcaHG* on the β-ketoadipate pathway (see Fig. 13).^109–111^ Titres of up to 50 g L^−1^ have been obtained from either *p*-coumaric acid or corn stover alkali pretreated lignin, which contains high levels of *p*-hydroxycinnamic acids,^109–111^ and the *cis,cis*-muconic acid bioproduct can then be chemically hydrogenated to yield adipic acid, used for manufacture of nylon.^109^ Muconic acid has also been generated in engineered strains of *Amycolatopsis* sp.,^112^ and *Corynebacterium glutamicum*, in the latter case in titres of 85 g L^−1^ from catechol, and 1.8 g L^−1^ from a hydrothermally depolymerised softwood lignin.^113^

Aromatic dicarboxylic acids have also been generated from engineered lignin-degrading strains, as potential replacements for terephthalic acid used in plastics manufacture. Insertion of *ligAB* or *praA* genes encoding protocatechuate 4,5-dioxygenase or protocatechuate 2,3-dioxygenase into *Rhodococcus jostii* RHA1 gave recombinant strains able to generate pyridine-2,4-dicarboxylic acid or pyridine-2,5-dicarboxylic acid respectively (see Fig. 13), from wheat straw lignocellulose, in titres of 106–125 mg L^−1^.^114^ In these cases the extradiol ring fission products generated are cyclised in *R. jostii* RHA1 with ammonium chloride present in M9 minimal media.^114^ Further engineering of *R. jostii* RHA1 *via* gene deletion of *pcaHG* genes on the β-ketoadipate pathway, chromosomal integration of *ligAB* genes, and overexpression of lignin-oxidising *dyp2*, gave a titre of 330 mg L^−1^ from wheat straw lignocellulose, and 240 mg L^−1^ from soda lignin.^115^ Pyridine-dicarboxylic acids have been successfully converted into PDCA-containing polymers, which show similar physical properties to conventional oil-based polymers containing terephthalic acid.^116^ Pyrone dicarboxylic acids have also been produced from lignin *via* protocatechuate degradation in *Novosphingobium aromaticivorans* DSM12444, *via* the pathway shown in Fig. 13.^117^ Perez *et al.* have knocked out *ligI*, *desC*, and *desD* genes in *N. aromaticivorans*, leading to accumulation of 0.49 mM 2-pyrone-dicarboxylic acid from a chemically depolymerised poplar lignin stream.^117^

4-Vinylguaiacol, an aroma chemical used in the food industry, has also been generated in engineered *Pseudomonas putida* KT2440 (see Fig. 13).^118^ Insertion of the *padC* gene encoding phenolic acid decarboxylase in place of the *ech* gene involved in ferulic acid metabolism gave a recombinant *P. putida* Δ*ech*::*padC* strain able to produce 62 mg L^−1^ 4-vinylguaiacol from a commercially available soda lignin.^118^

## 11. Conclusion

The reader will see that there are many challenges in breaking down lignin, but Nature has found elegant solutions to break difficult bond linkages, and solve problems of lignin repolymerisation and hydrogen peroxide generation. Enzyme combinations or enzyme cascades have the potential to harness these solutions to generate useful products from lignin breakdown, but still in mixtures of products, whereas engineered microbial lignin bioconversions can generate single products from lignin breakdown. The microbial pathways for lignin breakdown are still not fully understood, so there is scope for improvement in product titre from lignin, or these strategies could potentially be combined with chemocatalytic methods for lignin processing. Improvements in product titre will be needed in order to develop commercially viable routes from lignin to high-value products. Another challenge to commercialisation is that the technical lignins that are mainly produced by the pulp/paper industry are more difficult to convert, both chemically and biologically (see Section 6), so if that problem could be solved, then lignin producers could collaborate more effectively with research groups working on lignin conversion. Improved analytical methods for studying lignin structure are also helping to elucidate the molecular changes taking place during lignin breakdown,^48^ and improved analytical methods for studying lignin breakdown metabolites will help to elucidate mechanisms for lignin degradation.^102,103^

## Conflicts of interest

There are no conflicts of interest to declare.

## Supplementary Material
